# Reliability of a clinical method in estimating foetal weight and predicting route of delivery in term parturient monitored at a voluntary agency hospital in Southwest Nigeria

**DOI:** 10.4102/phcfm.v13i1.3017

**Published:** 2021-09-08

**Authors:** Temitope A. Yomibo-Sofolahan, Adekunle J. Ariba, Olusanya Abiodun, Ademola O. Egunjobi, Oluwaseun S. Ojo

**Affiliations:** 1 Olikoye Ransome Kuti Memorial Hospital, Asero, Abeokuta, Ogun State, Nigeria; 2 General Hospital Owode-Egba, Owode, Ogun State, Nigeria; 3 Department of Family Medicine, National Postgraduate Medical College of Nigeria, Ijanikin, Lagos, Nigeria; 4 Department of Family Medicine, Mercy Groups Clinics, Panseke, Abeokuta, Ogun State, Nigeria; 5 Faculty of Family Medicine, Olabisi Onabanjo University Teaching Hospital, Sagamu, Ogun State, Nigeria; 6 Society of Family Physicians of Nigeria, Nigeria; 7 Department of Obstetrics and Gynaecologist, Sacred Heart Hospital, Lantoro, Abeokuta, Ogun State, Nigeria; 8 Faculty of Obstetrics and Gynaecologist, West African College of Surgeons, Lagos, Nigeria; 9 Department of Obstetrics and Gynaecologist, Family Relations and Human Development, Nigeria; 10 Department of Family Medicine, Ogun State Hospital Management Board, General Hospital Ifo, Ifo, Ogun State, Nigeria; 11 Department of Family Medicine, Federal Medical Centre, Abeokuta, Ogun State, Nigeria

**Keywords:** estimated foetal weight, actual birth weight, Johnson’s formula, parturient, intrapartum

## Abstract

**Background:**

The antepartum estimation of foetal weight is a major determinant of the route of delivery and this has become vital in modern day obstetrics. The limitations to the use of obstetric ultrasonography, considered as the gold standard in estimating foetal weight, make clinical estimation methods attractive alternatives, especially in resource- constrained settings where many un-booked women may report for delivery.

**Aim:**

To determine the reliability of intrapartum clinical foetal weight estimation in predicting the actual birth weight (ABW) and route of delivery among term parturient.

**Setting:**

The study was conducted at the Sacred Heart Hospital, Lantoro, a voluntary mission agency hospital in Southwest Nigeria.

**Methods:**

This cross-sectional study was conducted among 291 term parturient recruited by systematic random sampling between June and September 2017. The clinical estimation of foetal weight was carried out using Johnson’s formula.

**Results:**

The accuracy of Johnson’s formula to predict the ABW was 59.5%; while for the mode of delivery, it was 130 (75.1%) for spontaneous vaginal delivery (SVD) and 43 (24.9%) for caesarean section (CS). The sensitivity of the accuracy of Johnson’s formula to predict the mode of delivery was 75.1%, with a specificity of 35.6%, a positive predictive value (PPV) of 63.1%, and a negative predictive value (NPV) of 49.4%.

**Conclusion:**

The intrapartum clinical foetal weight estimation at term determined by Johnson’s formula was reliably predictive of ABW and SVD, but it was unreliable in predicting the need for a CS.

## Introduction

Foetal weight and its estimation is a well-researched anthropometry because of its link to many health outcomes such as the perinatal, neonatal, infant and maternal mortality rates.^1,2^ These health indices reflect the health status of a country and constitute a major challenge for underdeveloped nations like Nigeria.^1,2^

Accurate and reliable foetal weight estimation is very vital in the third trimester when foetal growth assessment is most likely to influence clinical decisions in the management of labour and in determining the mode of delivery.^3,4,5^ Most term babies with weights in the normal range (2.5 kg – 4.0 kg) would be delivered vaginally, except when there are specific contraindications.^6^ Prior knowledge of the foetal weight also helps the clinician to be prepared to take measures and necessary interventions aimed at reducing the occurrence of foetal complications (such as shoulder dystocia, birth asphyxia, and skeletal injuries like fractures) as well as maternal complications (like prolonged labour, postpartum haemorrhage and severe maternal genital trauma) associated with delivery.^7,8^ At the primary care level, any un-booked parturient whose estimated foetal weight is adjudged too big for the birth canal is offered early referral to the appropriate healthcare level.^8,9^ Therefore, failure to estimate the likely value of the foetal weight as accurately as possible can lead to poor management and adverse outcome in labour and delivery. Hence, estimating foetal weight has become a universal and routine antenatal care practice in modern obstetric care.^3,6^

The main methods used for estimating foetal weight in current obstetric practice are imaging (ultrasonography and magnetic resonance imaging) and clinical techniques (abdominal palpation or Leopold’s manoeuvre, risk factors assessed clinically, maternal self-estimated foetal weight and the application of equations that predict birth weights).^1,3,4,5,6^

Obstetric ultrasonography is considered the gold standard in foetal weight estimations, but it is still not readily affordable and available, especially in low resource areas.^10,11^

Clinical (abdominal) palpation, also known as the tactile assessment or Leopold’s manoeuvre is easy to use, convenient and economical (affordable).^4^ However, it does not give the exact size or weight estimate of the baby; it only suggests that the baby may be too big for the mother’s birth canal.^3,10,11^ Maternal Self Estimated Foetal Weight uses some mothers’ own predictions of foetal weight based on their lifestyle and diet.^10^ However, there are no studies to dispel or confirm these notions.

Furthermore, various formulae and equations have been developed in estimating foetal weight.^3,6^ Majority of them are based on combining measured symphysio-fundal height (SFH) with some other parameters. Among these are:

Dare’s formula (Weight in Grams): written as:

FW=(AG×SFH)grams.^3,10,12^ [Eqn 1]

where, AG = abdominal girth (cm) and SFH = symphysiofundal height (cm);

Johnson’s formula written as foetal weight (FW):

$$(\text{g})=\text{SFH}(\text{cm})-n\times 155.^{3,6}\text{where, SFH}=\text{symphysiofundal height}$$ [Eqn 2]

and *n* is a constant whose value depends on the station (*n*).

*n* = 13 if the vertex is above the ischial spine (when the presenting part is not engaged), *n* = 12 if the vertex is at the level of the ischial spine (when the presenting part is at station 0), and *n* = 11 if the vertex is below the level of the ischial spine (when the presenting part is engaged);

Kongnyuy – Mbu’s formula written as:

$$\text{FW}=3{\left(\text{FH}\right)}^{2}$$ [Eqn 3]

where FW = foetal weight and FH is the fundal height^3^.

Dawn’s formula calculated as:

$$\text{EFW}=\text{longitudinal diameter of the uterus}\times (\text{transverse diameter})2\times 1.44/2.^{3}$$ [Eqn 4]

Algorithm derived from maternal and pregnancy-specific characteristics^3,6^ where:

Foetal weight(g)=gestational age(d)×[9.36+0.262×foetal sex+0.000237×maternal height(cm)×maternal weight at 26 weeks(kg)]+[4.81×maternal weight gain rate(kg/day)×(parity+1)]. [Eqn 5]

Where foetal sex is equal to +1 for a male foetus, −1 for a female foetus, and 0 if the sex is unknown.

In this study, clinical foetal weight estimations were carried out by using Johnson’s formula, which is easy to apply and considers both maternal and foetal characteristics.

The mode of delivery can either be a vaginal (vertex, breech or assisted vagina delivery) route or surgical (elective or emergency caesarean delivery) intervention.^8,13^ The choice of the route of delivery depends on the indication which may be maternal, foetal or fetomaternal.^14,15^ However, vaginal delivery is still the most preferred.^14,16^ Failure to correctly predict the most appropriate mode of delivery may result in avoidable morbidity for both mother and child, and sometimes death of either or both. Inaccurate estimation of foetal weight and inappropriate choice in the mode of delivery has led to an increase in perinatal and maternal mortality rates.^6,13^ Precise estimation of foetal weight will improve these health indices significantly, especially in low resource countries.

Therefore, the use of Johnson’s formula to estimate the foetal weight and predict the mode of delivery based on the foetal weight estimation will be relevant to the obstetrics caregiver at the primary care level and other peripheral centres. It will help them make a more formal and objective estimation of the foetal weight and thus predict the most likely mode of delivery. A prompt referral can also be made if the need arises, especially if the healthcare facility is not equipped to perform a caesarean delivery.

The study aims to determine the reliability of intrapartum clinical foetal weight estimation in predicting the actual birth weight (ABW) and route of delivery among term parturient.

The objectives of the study were to clinically estimate foetal weight using Johnson’s formula; compare the estimated foetal weight with the ABW; predict the mode of delivery based on the estimation of the foetal weight and compare the predicted mode of delivery with the actual mode of delivery; and lastly determine if the clinically estimated foetal weights are predictive of vaginal or caesarean deliveries among the selected study participants.

## Methods

The study was a cross sectional hospital-based study. It was carried out at the Sacred Heart Hospital (SHH), Lantoro, Abeokuta Ogun State, southwest geopolitical zones of Nigeria. This geopolitical zone is inhabited mainly by the Yoruba ethnic group, making Yoruba the major language. The other ethnic groups present are the Igbos, Hausas, Fulanis, and Ijaws, among others.^17^ The inhabitants are mostly traders, civil servants and self-employed businessmen and women.^18^ The SHH, Lantoro, was established in 1895. It is the oldest existing hospital in Nigeria with 300 beds. It provides healthcare to the people of Abeokuta and its environs.^19^ The Maternity Unit of the hospital is made up of antenatal and postnatal clinics, as well as antenatal, postnatal, labour wards, and a theatre. The unit provides basic and comprehensive essential obstetric care needed for the management of pregnancy, delivery and the postpartum period.

The study population consisted of 291 pregnant women who booked and delivered in the SHH after being admitted as active labour cases (either through spontaneous labour or by induction of labour) at term during the duration of the study. The gestational ages of the pregnancies were determined using the last menstrual period (LMP) or an obstetric ultrasound scan (USS) in the first trimester.

The inclusion criteria for enrolment into the study were term parturient (37 to 42 weeks of gestation) booked at the SHH Antenatal clinic (ANC) clinic with a single viable pregnancy in cephalic presentation and those in active labour with cervical dilatation of at least 4 cm. Those excluded were women with multiple gestations, mal-presentation, those booked for elective caesarean section, those who presented with complications in labour such as severe preeclampsia, eclampsia and antepartum haemorrhages, those with gestational diabetes, and parturient who presented with head-on-perineum or were in the second stage of labour.

The health workers in the ANC, antenatal and postnatal wards of the study centre were informed and sensitised about the study, including its aim and objectives. Their maximum cooperation was solicited. The participants were sensitised, familiarised and informed about the study right from the ANC; but the point of recruitment was the labour ward. A questionnaire was administered to each parturient who consented and met with the selection criteria. The details about the maternal socio-demographic data, obstetric, medical, family and social history were obtained from the case note and filled into the questionnaire.

Once recruited into the study, the parturient was assessed, starting with a quick general examination followed by the more specific obstetric examination. The maternal SFH was measured when there were no uterine contractions. It was taken with a non-elastic, non-stretchable tape calibrated in centimetres. With the patient in supine position and the bladder emptied, the SFH was measured with the tape measure (on the reverse side) from the highest point of the fundus of the uterus to the midpoint of the upper border of the symphysis pubis, and recorded to the nearest one decimal point. The station of the presenting part was determined during the vaginal examination. It is the lowermost portion of the presenting part of the foetus in relation to the maternal ischial spine. When it was at the level of the ischial spine, it was said to be at zero station, levels above the ischial spines are interpreted in negative values –1, –2, –3 stations, while levels below the ischial spines are interpreted in positive values +1, +2, +3.

The foetal weight was then estimated using the Johnson’s formula calculated as:

Foetal weight(g)=[SFH(cm)−n(where n can be 11,12, or 13 depending on the station of the presenting part)]×155. [Eqn 6]

If the maternal weight was more than 90 kg, 1 was subtracted from the SFH. On delivery of the baby, the appearance, pulse, grimace, activity and respiration (APGAR) score, and anthropometric parameters (weight, length, head, chest, abdominal circumference, and the mid-upper arm circumference) of the baby were taken. The ABW was taken within 30 min of delivery by the research assistant using a Way-master^®^ weighing scale with a maximum weight capacity of 10 kg. It was recorded to the nearest one decimal point. The research assistant who measured the ABW of the baby was blind to the estimated foetal weight carried out by the researcher, while the researcher was also blind to the ABW measurement and recording until the time to compare the results. This was to minimise bias.

Based on the obtained result from the clinically estimated foetal weight, a prediction of the mode of delivery (either spontaneous vaginal delivery [SVD] or cesarean section [CS]) was made and documented. The progress of the labour and the decision about the mode of delivery was left to the labour monitoring team of the hospital. The researcher had no contributions to these decisions.

A systematic random sampling method was used to achieve the desired sample size which was determined using the following formula for cross-sectional study:

$$(n=z^{2}pq/d^{2}).$$^20,21,22^ [Eqn 7]

*z* = the standard normal deviate often set at 1.96, which corresponds to 95% confidence interval (CI).

*p* = the expected prevalence/the proportion of patients with the attributes of interest.

*q* = 1−*p*,

*d* = the degree of accuracy required = 0.05.

The proportion of patients whose mode of delivery was based on predictions of the clinically estimated foetal weight was not available. Thus, *p* = 50%.

The minimum sample size for this study was, therefore, *n* = *z*^2^*pq*/*d*^2^

*n* = 1.962 × 0.5 × (1 – 0.5)/0.052

*n* = 3.84 × 0.5 × 0.5/0.0025

*n* = 384.

Correction for a total population less than 10 000 was done with the formula:

nf=n/1+(n)/(N).^20,21,22^ [Eqn 8]

Where nf = the desired sample size, when the population is less than 10 000.

*n* = the desired sample size, when the population is greater than 10 000.

*N* = the estimate of the population size.

The estimate of population size (*N*) was determined by multiplying the total number of term parturient with singleton foetus by the duration of study. From the records of the SHH, the average number of pregnant women who delivered annually was 3000, out of which 2580 (86%) of them were women who received ANC and delivered in the SHH Averagely, this translated to 2580 registered parturient annually, 215 monthly, 54 weekly, and 8 daily. The study lasted for 4 months.

Therefore, *N* = total number of parturient monthly × the duration of study.

N=215×4=860 parturient [Eqn 9]

Therefore, nf = 384

1+384/860 1.45=264.83 [Eqn 10]

Considering an attrition rate of 10% of the participants, the sample size:

- = (10% of 264.83) + 264.83
- = 26.483+264.83 = 291.31.

Hence, a total of 291 participants were recruited.

The data collection tool for this study was an interviewer-administered questionnaire, written in English language. It was designed from international standardised protocols, pretested and adapted accordingly to fit into the purpose of the study before final use. The primary outcome variable was the predictability of the accuracy of the clinical estimation of foetal weight using Johnson’s formula. The statistical analysis was done by using Statistical Package for Social Sciences (SPSS) version 21. The result of the study was analysed in different sub-groups using appropriate statistics. The socio-demographic data was expressed by using descriptive statistics. The quantitative variables (age, neonatal anthropometric parameters and placenta weight) were analysed using means and their standard deviations, while the qualitative variables (mode of delivery, sex) were analysed using frequencies and proportions. The birth weight was categorised into: low birth weight (LBW), normal birth weight (NBW), and macrosomia. Likewise, the mode of delivery was grouped into spontaneous vertex delivery and caesarean section. Chi-square was used to compare the weight (estimated and actual) and mode of delivery (predicted and actual), and also to determine their associations. The level of significance was designated as *p* = 0.05. The accuracy of Johnson’s formula in estimating the foetal weight was determined by calculating the proportion of estimates within 10% of ABW. The predictability of the clinically estimated foetal weight was determined by calculating the sensitivity, specificity, positive predictive value (PPV) and negative predictive value (NPV).

## Results

In this study, 299 booked parturient were recruited and monitored till delivery. The maternal ages ranged between 18 to 42 years, with a mean age of 29.7 ± 5.4 years. The minimum weight of the parturient in labour was 48 kg, while the maximum weight was 104 kg. The mean weight was 72.3 kg ± 10.6 kg. Seventeen (5.8%) of the parturient weighed more than 90 kg. Majority of the parturient, 202 (69.4%), were primigravida. At the point of recruitment, 229 (78.7%) of the mothers had cervical dilatation of at least 4 cm and 149 (51.2%) of the presenting part of the foetuses were at station zero (level of the ischial spine) (Table 1).

**TABLE 1:** Maternal characteristics of the parturient (*N* = 291).

Variables	*N*	%
**Age (years)**		
18–24	53	18.2
25–39	228	78.4
> 39	10	3.4
**Weight (kg)**		
Less than 90	274	94.2
Greater than 90	17	5.8
**Parity**		
Primigravida (1st pregnancy)	202	69.4
Multigravida (2nd to 4th pregnancy)	85	29.2
Grand multigravida (5th to 6th pregnancy)	3	1.0
Great-grand multigravida (7th pregnancy and above)	1	0.3
**Cervical dilatation**		
Active Labour (3 cm to 7 cm)	229	78.7
Transition (8 cm to 10 cm)	62	21.3
**Station at presentation**		
Above ischial spine (*n* = 13)	46	15.8
At ischial spine (*n* = 12)	149	51.2
Below ischial spine (*n* = 11)	96	33.0

The minimum ABW in this study was 2.3 kg and the maximum was 4.8 kg. The mean ABW was 3.2 kg ± 0.37 kg. The total number of clinically estimated foetal weight within accuracy of Johnson’s formula was 174 (59.8%) of which the LBW were 10 (3.4%), NBW were 157 (57.3%) and the macrosomic new-borns were 6 (85.7%) among the categories of birthweights. The error between clinically estimated foetal weight (EFW) and ABW was 0.70 kg ± 0.28 kg, the mean of error was 0.23 kg ± 0.12 kg, and the mean percentage error was 9.17% ± 6.13% (Table 2).

**TABLE 2:** Accuracy of Johnson’s formula among categories of birth weights.

Percentage accuracy of Johnson’s formula	Predictive value		ABW						*X*	*p*
			LBW		NBW		Macrosomia			
	*n*	%	*n*	%	*n*	%	*n*	%		
10%	173	59.5	10	100.0	157	57.3	6	85.7	9.350	0.009
Others	118	40.5	0	0.0	117	42.7	1	14.3		

**Total**	**291**	**100.0**	**10**	**3.4**	**274**	**94.2**	**7**	**2.4**	**-**	**-**

LBW, low birth weight; NBW, normal birth weight; ABW, actual birth weight; *X*, standard deviation; *p*, probability value.

A total of 206 (70.8%) parturient delivered SVD, 130 (75.1%) of them were within accuracy of Johnson’s formula. Also, a total of 85 (29.2%) parturient delivered through CS, and 43 (24.9%) of them were within accuracy of Johnson’s formula. There was no statistically significant relationship between the estimates within accuracy of Johnson’s formula and actual mode of delivery (*p* = 0.48) (Table 3).

**TABLE 3:** Estimates within accuracy of Johnson’s formula and their actual mode of delivery.

Actual mode of delivery	Predictive value		Percentage accuracy				*X*	*p* *
			Within 10%		Over 10%			
	*n*	%	*n*	%	*n*	%		
SVD	206	70.8	130	75.1	76	64.4	3.912	0.48
CS	85	29.2	43	24.9	42	35.6		

**Total**	**291**	**100.0**	**173**	**50.6**	**118**	**49.4**	**-**	**-**

SVD, spontaneous vaginal delivery; CS, caesarean section; *X*, standard deviation; *p*, probability value.

* , *p* = 0.05.

The determination of Johnson’s formula to predict vaginal or caesarean delivery (mode of delivery) was determined by calculating the sensitivity, specificity, PPV and NPV, which were 75.1%, 35.6%, 63.1% and 49.4%, respectively (Table 4).

**TABLE 4:** Johnson’s formula to predict the mode of delivery.

Outcome of predictive value	Positive (*n*)	Negative (*n*)	Total
Positive	True positive-Tp (130)	False positive-Fp (76)	Tp + Fp = 206
Negative	False negative-Fn (43)	True negative-Tn (42)	Fn + Tn = 85

**Total**	**173**	**118**	**291**

## Discussion

The parturient in this study were well educated, gainfully employed, relatively healthy and low parity mothers in the optimal years of their reproductive lives. These socio-demographic variables played a very significant influence on the optimal maternal and perinatal outcome obtained in this study.

The foetal weight clinical estimation was obtained by the application of Johnson’s formula, while the prediction of the mode of delivery was done based on the estimated foetal weight. Birth weight of 4.0 kg was used as the cut-off for foetal macrosomia. It was hypothesised that macrosomic foetuses of 4.0 kg and above would be delivered via CS, while those less than 4.0 kg were predicted to be delivered via SVD. It is believed that maternal obesity can lead to over estimation of foetal weight possibly because of the presence of increased abdominal fat.^3,5^ Johnson’s formula made an adjustment based on maternal obesity by subtracting one (1) from the SFH if the maternal weight was more than 90.0 kg.^3,5,11^

The mean clinically estimated feotal weight (CEFW) in this study was higher than the 3.5 kg in a south-south Nigerian study by Njoku et al. on estimating foetal weight.^11^ The mean ABW of this study was comparably similar to 3.2 kg reported by Shittu et al. in Ile-Ife,^23^ and 3.2 kg reported by Njoku et al. in Calabar.^11^ It is also comparable to 3.2 kg reported in South Africa. The mean ABW was also comparably close to 3.1 kg ± 0.39 kg and 3.33 kg ± 0.4 kg reported in Borden Guard Hospital, India^24^ and in a State hospital in Turkey, respectively.^5^ It was observed in this study that although Johnson’s formula overestimated foetal weight, there was a statistically significant relationship between clinically estimated foetal weight using Johnson’s formula and the ABW, with a *p*-value of 0.001. The accuracy of the CEFW using Johnson’s formula was determined by calculating the estimates within 10% accuracy of ABW.^9,2,25^ The estimates within accuracy of Johnson’s formula captured all categories of birth weights with higher percentage accuracy for extremes of birth weights. There was a statistically significant relationship between the CEFW within 10% of accuracy and ABW (*p* = 0.009). In addition, the mode of delivery for estimates within accuracy of Johnson’s formula was reliably predictive of SVD, but not for CS.

## Conclusion

This study further confirms the ability of Johnson’s formula to reliably predict the ABWs of new-borns even when their mothers are encountered for the first time in established labour. Such CEFW however, can only be used to predict normal vaginal delivery and not caesarean delivery. This implies that even if the birth weight can almost be accurately predicted during labour, several other factors such as intrapartum bleeding, foetal and maternal distress can come to play and alter the mode of delivery. Such unforeseen and unpredictable circumstances are worthy of consideration in determining the mode of delivery.

In summary, our findings in this study have important clinical relevance especially in low resource areas where there are constraints to the availability and use of ultrasonography. The relationship between clinically EFW in labour and mode of delivery in term parturient is such that the clinically estimated foetal weight is more predictive of SVD but not of CS. Based on these, the clinically estimated foetal weight cannot be generalised to predict the mode of delivery.
